# Berberine in the Treatment of Type 2 Diabetes Mellitus: A Systemic Review and Meta-Analysis

**DOI:** 10.1155/2012/591654

**Published:** 2012-10-15

**Authors:** Hui Dong, Nan Wang, Li Zhao, Fuer Lu

**Affiliations:** ^1^Institute of Integrated Traditional Chinese and Western Medicine, Tongji Hospital, Tongji Medical College, Huazhong University of Science and Technology, Wuhan, Hubei 430030, China; ^2^Department of Radiology, Tongji Hospital, Tongji Medical College, Huazhong University of Science and Technology, Wuhan, Hubei 430030, China

## Abstract

*Objectives.* To assess the efficacy and safety of berberine in the treatment of type 2 diabetes mellitus (T2DM). *Methods.* Randomized trials of berberine compared with lifestyle modification, placebo, and/or oral hypoglycaemics intervention on treating T2DM were included. Study population characteristics and outcome results were extracted independently by two reviewers. Meta-analyses were performed for data available. *Results.* Fourteen randomized trials, involving 1068 participants, were included in this study. Methodological quality was generally low. Compared with lifestyle modification with or without placebo, the cointervention of berberine and lifestyle modification showed significantly hypoglycaemic and antidyslipidemic response. Compared with oral hypoglycaemics including metformin, glipizide, or rosiglitazone, berberine did not demonstrate a significantly better glycaemic control but showed a mild antidyslipidemic effect. Compared with oral hypoglycaemic drugs, cointerventions with berberine and the same oral hypoglycaemics showed a better glycaemic control. No serious adverse effects from berberine were reported. *Conclusions.* Berberine appeared to be efficacious for treating hyperglycaemia and dyslipidemia in T2DM. However, the evidence of berberine for treating T2DM should be carefully interpreted due to the low methodological quality, small sample size, limited number of trials, and unidentified risks of bias.

## 1. Introduction

The prevalence of diabetes mellitus (DM) has continued to increase globally. According to the latest figures from the International Diabetes Federation (IDF), the number of individuals with diabetes in 2011 has reached a staggering 366 million, causing 4.6 million deaths each year. Type 2 diabetes mellitus (T2DM) is the most common form of diabetes. Initial therapy for treating T2DM includes diet and exercise, followed by the use of oral hypoglycemic agents and potentially subcutaneous insulin injections [1, 2]. Evidence from many multicenter trials has demonstrated that the above pharmaceuticals are able to lower blood glucose and to reduce the risk of developing diabetic complications. However, there are also a number of limitations of currently available antidiabetic drugs. For example, the treatment with metformin is associated with a high incidence of gastrointestinal side effects [3]. Thiazolidinediones have the potential to increase the risk of cardiovascular disease. Those adverse effects limit their widespread use in clinical practice [4]. 

Consequently, many diabetic patients are also suggested to receive complementary and alternative medicine therapies. This is particularly true in China [5]. DM is referred to as “Xiao Ke” disease (which means emaciation and thirst) in Chinese medicine, which is a consequence of over-intake of greasy food and sedentary lifestyle [6]. There is a long history of using herbal medications to treat diabetes in China. Numerous researches also suggest that some herbal therapies may have a role in the treatment of this complex disease [7]. Among effective herbs, Rhizoma Coptidis (Huang Lian) and its major constituent berberine attract much attention for their glucose-lowering activities by many reports [8–11]. 

Berberine is an isoquinoline derivative alkaloid isolated from Rhizoma Coptidis, which has been widely used as a drug to treat gastrointestinal infections (e.g., bacterial diarrhea). The content of berberine in Rhizoma Coptidis is about 5.2% to 7.7%. The hypoglycemic effect of berberine was first reported in 1988 when it was used to treat diarrhea in diabetic patients [22]. Since then, berberine has been used as an antihyperglycemic agent by many physicians in China. A number of clinical trials have also been reported on this subject in medical journals during the past 30 years. 

Meanwhile, many studies have been devoted to elucidate the molecular mechanisms underlying the hypoglycaemic effect of berberine. The key findings support that the antidiabetic actions of berberine include increasing secretion of insulin, improving insulin resistance, and ameliorating dyslipidemia [23–25]. The hypoglycaemic effect of berberine is also partially mediated by an anti-inflammatory mechanism, which adds new evidence indicating that type 2 diabetes mellitus is a low-grade inflammatory disease [26].

While berberine is effective on improving hyperglycaemia, there exist a number of issues. At this point, there are few multicenter clinical trials to confirm the hypoglycaemic action in a larger number of patients. Additionally, the scientific evidence that berberine is as effective as other conventional treatments in treating T2DM remains to be further validated. In terms of safety concerns, it is also not sure about the safety of a long-term berberine intake for the chronicity of diabetes. This concern has been raised since the initial application of berberine as an antidiarrhea agent, as well as the reports of berberine-induced haemolysis in chronic haematological diseases [27]. Considering this, it is necessary to assess the current trials to systematically review the potential role and safety for long-term use of berberine in the treatment of T2DM.

## 2. Materials and Methods

### 2.1. Search Strategy

We searched the following electronic databases for the identification of trials: MEDLINE, EMBASE, the Cochrane Library, China Academic Journal Network Publishing Database (CAJD), Wanfang database, China Doctoral Dissertations Full-text Database (CDFD), China Master's Theses Full-text Database (CMFD), and Chinese Academic Conference Papers (CACP). Databases of ongoing trials were also searched. All the above databases were searched from the available date of inception until the latest issue (March 2012). No language restriction was used. 

We combined different search strategies as follows: for English databases we use free text terms as berberine and diabetes; for Chinese databases we use free text terms as “Huang Lian Su” or “Xiao Bo Jian” (which is the alternative name of berberine in Chinese), and “Tang Niao Bing” or “Xiao Ke (which means diabetes in Chinese). A filter for clinical trials was applied. We also attempted to identify additional studies by searching the reference lists of included trials.

### 2.2. Selection Criteria

Randomized clinical trials (RCTs) were included irrespective of blinding, publication status, or language. People with T2DM, preexisting or newly diagnosed, were included. To be consistent with changes in diagnostic criteria of T2DM through the years, the diagnosis should have been established using the diagnostic criteria valid at the time of the beginning of the trial [28]. For the types of interventions, treatments with berberine, alone or combined with lifestyle modification and oral hypoglycaemics (e.g., metformin, insulin secretagogues, acarbose, thiazolidinediones), in RCTs were considered. The control intervention included lifestyle modification, placebo and the same cointervention of antidiabetic agents. Some studies contained multiple groups and each group was considered as a separate study in the analysis. Trials were only included if the intervention was given for at least eight weeks. 

We excluded case reports, studies without a control group, or any active intervention used with herbal medicines, acupuncture, and other pharmacological compounds. For obvious duplicate studies, authors of reports were contacted to clarify uncertainty. If the author could not be contacted, the first published report was regarded as the original. Intervention with insulin was also excluded because it was unreasonable for not changing insulin doses during an eight-week period. RCTs without a clear description of plasma glucose levels, particularly those not describing exact means and standardized deviations of glucose, were also excluded from our analysis.

### 2.3. Data Extraction and Management

Two reviewers (Dong and Wang) independently assessed trials for inclusion in the review. They extracted data concerning details of the sample size, interventions, duration of treatment, and outcomes by using a standard data extraction template. Any disagreements were resolved by consensus, or if required by a third reviewer (Lu). Risk of bias was assessed for the following criteria: baseline difference, method of randomization, allocation concealment, blinding, loss of participants and intention-to-treat (ITT) analysis, and followup. Studies meeting most of these criteria were regarded as high-to-moderate quality. 

The primary outcomes consisted of fasting plasma glucose levels (FPG), postprandial plasma glucose levels (PPG), glycosylated haemoglobin levels A1c (HbA1c), and adverse effects. The secondary outcomes consisted of fasting insulin levels (FINS) and plasma lipids which included triglycerides (TG), total cholesterol (TC), high-density lipoprotein cholesterol (HDL-C), and low-density lipoprotein cholesterol (LDL-C). Where outcomes were ambiguous or missing in the article, the author was contacted. If the author could not be contacted, the decision to extract the data was resolved by consensus. 

### 2.4. Data Synthesis and Analysis

To summarize the effects of berberine, we used the Review Manager 5.1 meta-analysis software to calculate weighted mean differences (MD), standard mean differences (SMD), and the 95% confidence interval (CI) for continuous data. MD was used if outcomes were measured in the same way between trials for continuous data while SMD was used to combine trials that measured the same outcomes, but used different methods. The heterogeneity was evaluated with the chi-square test, tau^2^ test, and the Higgins *I* ^2^ test. The different berberine interventions and control methods were used for sensitivity subgroup analyses. Reporting bias was explored through funnel plot analysis when the number of included trials exceeded five. A fixed-effect model was used when the studies in the subgroup were sufficiently similar (*P* > 0.10). Otherwise, a random-effects model was used. The overall effect was tested by using *Z* score with significance being set at *P* < 0.05.

Since the different berberine interventions and control methods may lead to substantial clinical heterogeneity, the study results of included trials were not combined. Alternatively, we performed the following three subgroup analyses in order to minimize the heterogeneity: berberine with a cointervention of lifestyle modification versus a control of lifestyle modification alone or plus placebo; berberine versus oral hypoglycaemics; berberine combined with oral hypoglycaemics versus the same oral hypoglycaemics alone.

## 3. Results

Seventeen RCTs involving berberine and T2DM were identified. Three trials were excluded: Yu et al. [29], Rong et al. [30], and Qiu et al. [31]. Although Yu et al. claimed that they conducted a randomized-control trial, tremendous difference in the number of patients was found between two-armed parallel groups. For the same reason the study of Rong et al. [30] was excluded. The uncertain description of using the oral hypoglycaemics in the control group was the reason why the study of Qiu et al. [31] was excluded. Fourteen RCTs met our inclusion criteria, and the details of the trials were listed in Tables 1 and 2. Together, those trials included a total of 1068 patients.

### 3.1. Studies Description

The included studies were published as full text between 2007 and 2011. All RCTs originated from China. Three studies were published in English [9–11] and the remaining eleven studies were published in Chinese [12–21, 32]. Thirteen of the fourteen trials were performed as single center trials while one study was a multicenter trial. 

As presented in Table 1, nine trials adopted a two-armed parallel group design. Five trials adopted a three- or four-armed group design which was shown in Table 2. Li and Liu [20] designed three parallel groups, which were glipizide, berberine, and the combination of both drugs. Three parallel groups were also found in the trial of Li [13], which were metformin, berberine, and the combination of both interventions. Three parallel groups were included in the design of Xiang et al. [21], which were lifestyle modification plus placebo, lifestyle modification with aspirin, and lifestyle modification with berberine. Three parallel groups were included in the trial of Zhang et al. [11], which were berberine, metformin, and rosiglitazone. There were four parallel groups in the design of Cao [19], and the interventions were lifestyle modification alone, lifestyle modification with metformin, lifestyle modification with berberine, lifestyle modification with berberine, and Qihuang capsule (which contains some types of herbs), respectively. 

### 3.2. Intervention and Controls

 Four studies randomized participants to receive berberine with a cointervention of lifestyle modification versus a control of lifestyle modification alone or plus placebo. Seven trials compared berberine with one kind of oral hypoglycaemic drugs (metformin, glipizide, rosiglitazone). Four trials compared a cointervention of berberine and one type of oral hypoglycaemics (metformin, glipizide) with a control of the same hypoglycaemics. Two trials compared a cointervention of berberine and two types of oral hypoglycaemics (metformin, glipizide, or glimepiride) with a control of the same hypoglycaemics. 

The dose of berberine used in the included trials was different. Berberine intake was generally in a range between 0.5 g and 1.5 g per day. The total daily berberine intake was divided into two or three doses. However, in the article of Zhang et al. [17], the dose of berberine was 20 mg per kilogram for each participant. One trial used three kinds of doses of berberine based on FPG levels of the participants at the time of inclusion [12]. The dose was stable and remained unchanged during the period of study in twelve trials. Two trials reduced the dose of berberine during the period of study when the gastrointestinal discomfort occurred [9, 14]. 

The duration of interventions in the included trials was also different, ranging from eight to twenty-four weeks. The interventions lasted for eight weeks in three trials, while twelve weeks or thirteen weeks in ten trials. In the trial of Yin et al. [18] the patients had received the intervention for 24 weeks.

### 3.3. Objectives and Outcomes

All the included trials were performed to evaluate hypoglycaemic and/or anti-dyslipidemic effects of berberine. The outcomes reported were mainly surrogate parameters including blood glucose, HbA1c, FINS, and plasma lipids. Adverse effects were reported in eleven trials. All the reported outcomes were measured at the end of the intervention. Six trials showed the primary and the secondary outcomes completely. The rest trials only presented a part of the outcomes. Thirteen trials performed treated-per-protocol (TPP) analysis, and one performed ITT analysis. 

With the assistance of a statistician, the outcomes in metformin and rosiglitazone groups in the trial of Zhang et al. [11] were combined. Cao [19] set the intervention in the fourth group as berberine and Qihuang capsule, and thus was not included due to not meeting the inclusion criteria. For the same reason, the outcomes of aspirin group in the study of Xiang et al. [21] were not included. Cointervention of anti-dyslipidemic agents was used in the trial of Zhang et al. [17] and the outcomes of plasma lipids were also not included. The reason we discarded the outcomes of plasma lipids in the trial of Yin et al. [18] was the data similarity of TC and LDL-C. 

### 3.4. Quality of the Included Studies

Most of the included trials in this meta-analysis were of poor quality, as indicated by unclear random sequence generation, inadequate allocation concealment, inadequate blinding, and undescribed withdrawal or dropouts (JADAD score ≤ 3), suggesting high risk of bias. Only one trial [9] performed a randomized, double-blind, placebo-controlled trial in four centers. Randomization was also performed centrally and was concealed and stratified in blocks of four. Although we performed three subgroups analysis to minimize the clinical heterogeneity, other potential sources of bias in the included studies were still significant. For example, the variation in the dose, duration, and type of the interventions might have contributed to the clinical heterogeneity among the studies. In addition, the methodological heterogeneity may arise through the diversity of results, which was attributed to different laboratory methods for the parameters determinations. 

Funnel plot analysis showed that there were no significant publication biases for the comparisons (numbers of included trials > 5). However, these cannot be considered reliable as there were fewer than 10 trials. It has been reported that the power of funnel plot is limited unless substantial bias is present and the number of trials is 10 or more [33]. In addition, funnel plot asymmetry may occur by chance.

### 3.5. Effects of Interventions

#### 3.5.1. Berberine with a Cointervention of Lifestyle Modification versus a Control of Lifestyle Modification Alone or Plus Placebo

Four trials (involving 271 patients) evaluated the therapeutic effect of berberine with a cointervention of lifestyle modification versus a control of lifestyle modification in the presence or not of placebo [9, 14, 19, 21]. Zhang et al. [9] and Xiang et al. [21] used placebo as the control. The number of trial participants ranged from 20 to 58 participants, with the trial duration of 12 weeks. As shown in Table 3, the statistical heterogeneity between the studies was significant among the results of FINS, TC, and LDL-C (*P* < 0.10). Pooled results showed a significant difference between berberine-treated group and the control group. Berberine with a cointervention of lifestyle modification was better than lifestyle modification alone or plus placebo in terms of improving FPG (*P *< 0.00001; MD −0.87 mmol/L; 95% CI −1.20 to −0.54), PPG (*P*  <  0.00001; MD −1.72 mmol/L; 95% CI −2.32 to −1.11), and HbA1c (*P* < 0.00001; MD −0.72%; 95% CI −0.97 to −0.47). Meanwhile, plasma levels of TG (*P* < 0.00001; MD −0.48 mmol/L; 95% CI −0.57 to −0.39) and LDL-C (*P* < 0.00001; MD −0.58 mmol/L; 95% CI −0.78 to −0.39) were significantly decreased and HDL-C (*P* < 0.0001; MD 0.07 mmol/L; 95% CI 0.04 to 0.10) levels were increased. To a smaller extent, plasma levels of TC (*P* = 0.01; MD −0.58 mmol/L; 95% CI −1.02 to −0.14) and FINS (*P* = 0.04; SMD −0.50 mU/L; 95% CI −0.96 to −0.03) were decreased in berberine with a cointervention of lifestyle modification group. 

#### 3.5.2. Berberine versus Oral Hypoglycaemics

Seven trials (involving 448 patients) compared berberine with oral hypoglycaemics [10, 11, 13, 19, 20, 25, 32]. The number of trial participants ranged from 15 to 51 with trial duration ranging from 8 to 13 weeks. In the pool of results on metabolic measures there was remarkable statistical heterogeneity among the comparisons, particularly PPG, FINS, TG, and HDL-C (*P* < 0.00001). No meta-analysis was conducted due to considerable statistical heterogeneity. Therefore, we described the outcomes of the trials included separately.

Three trials [13, 20, 32] did not report the difference in PPG levels between the berberine and control groups. One trial [19] showed that berberine was worse than the control in terms of reducing PPG levels, whereas one trial [10] showed that berberine appeared to be better than the control group with regard to reducing PPG levels.

Two trials [20, 32] did not report the difference in FINS levels between the berberine and control groups. One trial [19] showed that berberine was worse than the control in terms of reducing FINS levels. In contrast, one trial [10] showed that berberine has a better efficacy than the control in terms of reducing FINS levels.

Two trials [20, 32] did not compare TG levels in the berberine group with those in the control group. Two trials [10, 11] reported that berberine was better than the control in terms of reducing TG levels. However, one trial [19] showed that there was no significant difference in TG outcomes between the berberine and control groups. 

No data were available for differences in HDL-C levels between the berberine and control groups in two trials [20, 32]. Two other trials [10, 19] reported that there was no significant difference in HLD-C outcomes between the berberine and control groups. 

As shown in Table 4, there was no significant difference between berberine and oral hypoglycaemics in terms of reducing FPG (*P* = 0.21; MD 0.20 mmol/L; 95% CI −0.11 to 0.51) and HbA1c (*P* = 0.28; MD −0.11%; 95% CI −0.32 to 0.09). However, compared with those taking oral hypoglyceamics, patients who took berberine showed significantly better results of TC (*P* < 0.0001; MD −0.40 mmol/L; 95% CI −0.59 to −0.20) and LDL-C (*P* = 0.04; MD −0.33 mmol/L; 95% CI −0.65 to −0.01). 

#### 3.5.3. Berberine Combined with Oral Hypoglycaemics versus the Same Oral Hypoglycaemics Alone

Six trials (involving 396 patients) compared a cointervention of berberine and oral hypoglycaemics with the same oral hypoglycaemics alone [12, 13, 15, 16, 18, 20]. The number of trial participants ranged from 17 to 51 with the trial duration ranged from 8 to 24 weeks. As shown in Table 5, the statistical heterogeneity between the studies was significant in the results of PPG, HbA1c, FINS, TG, and LDL-C (*P* < 0.10). There was a significant improvement in FPG (*P* < 0.00001; MD −0.59 mmol/L; 95% CI −0.83 to −0.35), PPG (*P* = 0.0003; MD −1.05 mmol/L; 95% CI −1.62 to −0.48), HbA1c (*P* = 0.01; MD −0.53%; 95% CI −0.95 to −0.11), and FINS (*P* = 0.004; SMD −0.84 mU/L; 95% CI −1.42 to −0.26) in patients who took berberine and oral hypoglycaemics. In two of these trials [16, 20], combination of berberine and oral hypoglycaemics did not significantly improve TG (*P* = 0.36; MD −0.22 mmol/L; 95% CI −0.69 to 0.25), LDL-C (*P* = 0.29; MD −0.60 mmol/L; 95% CI −1.72 to 0.52) and HDL-C (*P* = 0.60; MD 0.02 mmol/L; 95% CI −0.06 to −0.11) levels compared with the control group. Plasma TC levels (*P* = 0.01; MD −0.47 mmol/L; 95% CI −0.83 to −0.11) were moderately reduced after the addition of berberine intervention.

### 3.6. Adverse Effects

Eleven of fourteen trials reported outcomes for adverse effects whereas the rest three reported no adverse effects during the berberine treatment [10, 11, 21]. Three of these reported the incidence of abdominal discomfort, but did not report the group in which abdominal discomfort occurred [13, 15, 18]. Five trials mentioned in detail that the adverse effects occurred in the berberine intervention group [9, 14, 16, 19, 20]. In the trial of Cao [19], there were seven incidences of abdominal discomfort like nausea, abdominal distension, and diarrhea, which were all from the berberine group. The symptoms were relieved after taking postprandial berberine treatments. Li and Liu [20] reported a few patients who developed a mild diarrhea caused by the intake of berberine. In the trial of Wang [14], one incidence of constipation occurred and was relieved after reducing the dose of berberine to 0.2 g three times a day. There were four dropouts in the trial of Zhang et al. [9], three from the placebo group and one from the berberine group due to losing followup. They also reported mild-to-moderate constipation that occurred in five participants who took berberine. Constipation in three participants in the berberine group was relieved without dose reduction, and two patients with mild constipation reduced berberine dose to 0.25 g twice daily. In the trial of Ye [16], the incidence of tolerable mild constipation occurred and the dose of berberine did not change during the period of study. No severe hypoglycemia was observed in all the included trials. 

There was no significant difference between the berberine and the control groups regarding the incidence of adverse effects. No serious adverse events were observed.

## 4. Discussion

Although many clinical trials regarding the antidiabetic effect of berberine have been conducted a systemic review was firstly reported by Na et al. in January 2012 [34]. The author also published the same abstract in the supplementary issue of Diabetes [35]. Ten RCTs involving 647 Chinese patients with T2DM were included in their review. However, no meta-analysis was performed due to significant statistical heterogeneity. Thus, the author only reported the outcomes of each trial. Compared with the previous review, we added another four studies: Liu and Hu [12], Zhang et al. [11], Ye [16], and Xiang et al. [21]. We also set three subgroups in order to minimize the heterogeneity, which resulted in combing the data successfully and performing a meta-analysis. For these reasons, our systemic review differs from the previous one. 

Unlike the previous review, our review indicates that berberine with lifestyle modification was more effective in terms of lowering FBG compared with lifestyle intervention alone or plus placebo. Our review also suggests that berberine has a much better effect on reducing PPG and HbA1c levels. With regard to lowering glucose and HbA1c (shown in Table 3), berberine appeared to improve blood glucose control in terms of normalization or an obvious reduction. The similar glycaemic control was observed when berberine was compared with the conventional antidiabetic therapy. Furthermore, berberine showed additional hypoglycaemic effect when combined with the antidiabetic agents. These outcomes suggest that berberine has a potential hypoglycaemic effect, which seems to be as effective as the conventional oral hypoglycaemics.

Our review also showed that some of the plasma lipid profiles in diabetic patients were improved by the berberine intake during a two- or three-month period. But the outcomes were various regarding the different comparisons of three subgroups. When berberine and lifestyle modification were compared with lifestyle modification alone or plus placebo, plasma levels of TG, TC, and LDL-C were decreased and HDL-C was increased, indicating an additional effect on dyslipidemia. However, compared with oral hypoglyceamics, those taking berberine just showed better results for TC, and LDL-C without any effect on HDL-C. The result of plasma TG level was controversial. Compared with oral hypoglycaemics alone, the cointervention of berberine and oral hypoglycaemics did not affect plasma levels of TG, LDL-C and HDL-C while a moderate reduction of TC level was observed. Generally, berberine appeared to have an additional cholesterol-lowering effect in treating diabetes, which was also found in the patients with hyperlipidemia [36, 37]. However, in different subgroup comparisons berberine, showed a different effect on TG, LDL-C, and HDL-C levels. This inconsistency may be associated with the limited number, small sample size, and heterogeneity of the included trials. It was also the reason for controversial outcomes of FINS after the intervention of berberine. Therefore the additional effect of berberine on dyslipidemia and plasma insulin level, which was rarely reported in conventional hypoglycaemics, should be more carefully assessed. 

In addition, berberine evaluated in our review generally appeared to be safe. The side effects were commonly gastrointestinal discomforts including constipation, diarrhea, nausea, and abdominal distension. Constipation was one of the most common gastrointestinal complaints among diabetic patients after berberine intake. It was a predictable side effect since berberine had a long history used as a remedy for diarrhea in China. But it was tolerable and relieved after reducing the dose of berberine. No severe hypoglycemia was found in the included trials. 

This systematic review also has several limitations. First, all trials included were conducted among Chinese participants in the mainland of China. There was a high risk of selection bias. We were not sure if the results were valid and applicable to other ethnic origin. Second, most of the studies were of poor quality. Only one study [9] was double-blinded and performed adequate allocation concealment. Two studies [12, 32] did not use blinding but performed unclear allocation concealment. The remaining eleven studies did not use blinding and allocation concealment. Thus, potential bias in selection of patients, administration of treatment, and assessment of outcomes could lead to overestimation of the therapeutic efficacy of berberine. Third, the limited number (from 4 to 7) of the trials included in each subgroup constrained the positive evidence of berberine for diabetes. During data extraction, we also found that only two or three studies provided the available data, especially for the outcomes related to plasma lipids. Quantitative subgroup analyses should not be performed when lacking insufficient data. The latter was also the reason why we were not able to draw a solid conclusion about the efficacy of berberine on dyslipidemia. Lastly, the heterogeneity between the trials included in each subgroup was also significant. It arose though the differences in the type of control method, dose of treatment, and duration of intervention of the included studies. The variation of participant age, gender, and blood glucose level at baseline may have additionally contributed to heterogeneous results. Therefore all of the outcomes should be carefully interpreted based on substantial methodological and clinical diversity.

## 5. Conclusion

Based on the existing evidence reviewed, berberine has beneficial effects on blood glucose control in the treatment of type 2 diabetic patients and exhibits efficacy comparable with that of conventional oral hypoglycaemics. The anti-dyslipidemic effect of berberine need to be further confirmed. Additionally, it has no serious adverse effects except for a mild to moderate gastrointestinal discomfort. Due to the lack of high quality clinical trials, the efficacy of berberine at treating diabetes remains to be validated. This is particularly true for the effect of berberine on improving dyslipidemia in T2DM. As such, large and well-designed randomized controlled trials should be performed before we recommend berberine for routine clinical use as an effective agent against T2DM. 

## Figures and Tables

**Table 1:** Characteristics of included trials (two-armed parallel group).

Author	Number of patients		Intervention			
	Experimental	Control	Experimental	Control	Duration (wks)	Outcomes
Liu and Hu 2008 [12]	30	30	LM, Ber, Met	LM + Met	8	FBG, PPG, HbA1c, FINS
Li 2008 [32]	33	32	LM, Ber	LM + Met	12	FBG, PPG, HbA1c, FINS, TC, TG, LDL-C, HDL-C
Wang 2008 [14]	30	31	LM, Ber	LM	12	FBG, PPG, HbA1c, FINS, TC, TG, LDL-C, HDL-C, AE
Zhang et al. 2008 [9]	58	52	LM, Ber	Placebo, LM	12	FBG, PPG, HbA1c, FINS, TC, TG, LDL-C, HDL-C, AE
Yin et al. 2008 [10]	15	16	LM, Ber	Met, LM	13	FBG, PPG, HbA1c, FINS, TC, TG, LDL-C, HDL-C, AE
Sheng and Xie 2010 [15]	30	30	Ber, Met, Glip	Met, Glip	12	FBG, FINS, AE
Ye 2010 [16]	40	40	Ber, Met, Glim	Met, Glim	12	FBG, PPG, HbA1c, TC, TG, LDL-C, HDL-C, AE
Zhang et al. 2011 [17]	30	30	LM, Ber	LM, Ber, Ros	12	FBG, HbA1c, TC, TG, LDL-C, HDL-C
Yin et al. 2011 [18]	30	30	LM, Ber, Met	LM, Met	24	FBG, PPG, HbA1c, FINS, TC, TG, LDL-C, HDL-C, AE

Note: AE: adverse effect; Ber: berberine; Glim: glimepiride; Glip: glipizide; LM: lifestyle modification; Met: metformin; Ros: rosiglitazone.

**Table 2:** Characteristics of included trials (three-armed and four-armed parallel group).

Author	Number of patients in each group				Intervention in each group					
	1	2	3	4	1	2	3	4	Duration (wks)	Outcomes
Cao 2007 [19]	30	30	30	30	LM	LM, Ber	LM, Ber, Qi	LM, Met	12	FBG, PPG, HbA1c, FINS, TC, TG, LDL-C, HDL-C, AE
Li and Liu 2007 [20]	51	51	50		Ber	Ber, Glip	Glip		8	FBG, PPG, HbA1c, FINS, TC, TG, LDL-C, HDL-C, AE
Li 2008 [13]	17	18	17		Ber	Ber, Met	Met		12	FBG, PPG, AE
Zhang et al. 2010 [11]	50	26	21		Ber	Met	Ros		8	FBG, HbA1c, TG, AE
Xiang et al. 2011 [21]	20	20	20		LM	LM, Ber	LM, Asp		12	FBG, PPG, HbA1c, TC, TG, LDL-C, AE

Note: AE: adverse effect; Asp: aspirin; Ber: berberine; Glip: glipizide; LM: lifestyle modification; Met: metformin; Qi: Qihuang capsule.

**Table 3:** Berberine with lifestyle modification versus lifestyle modification alone or plus placebo.

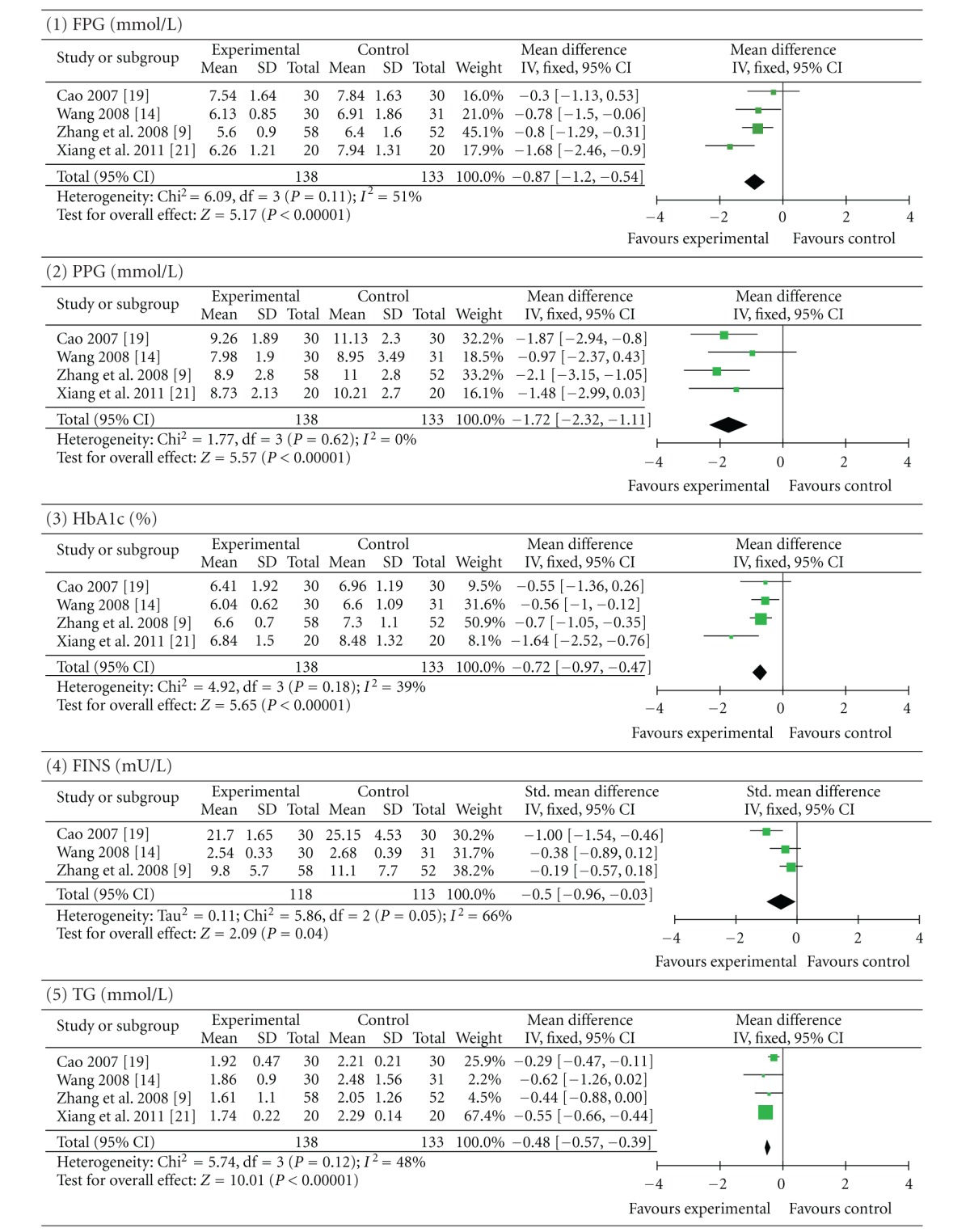 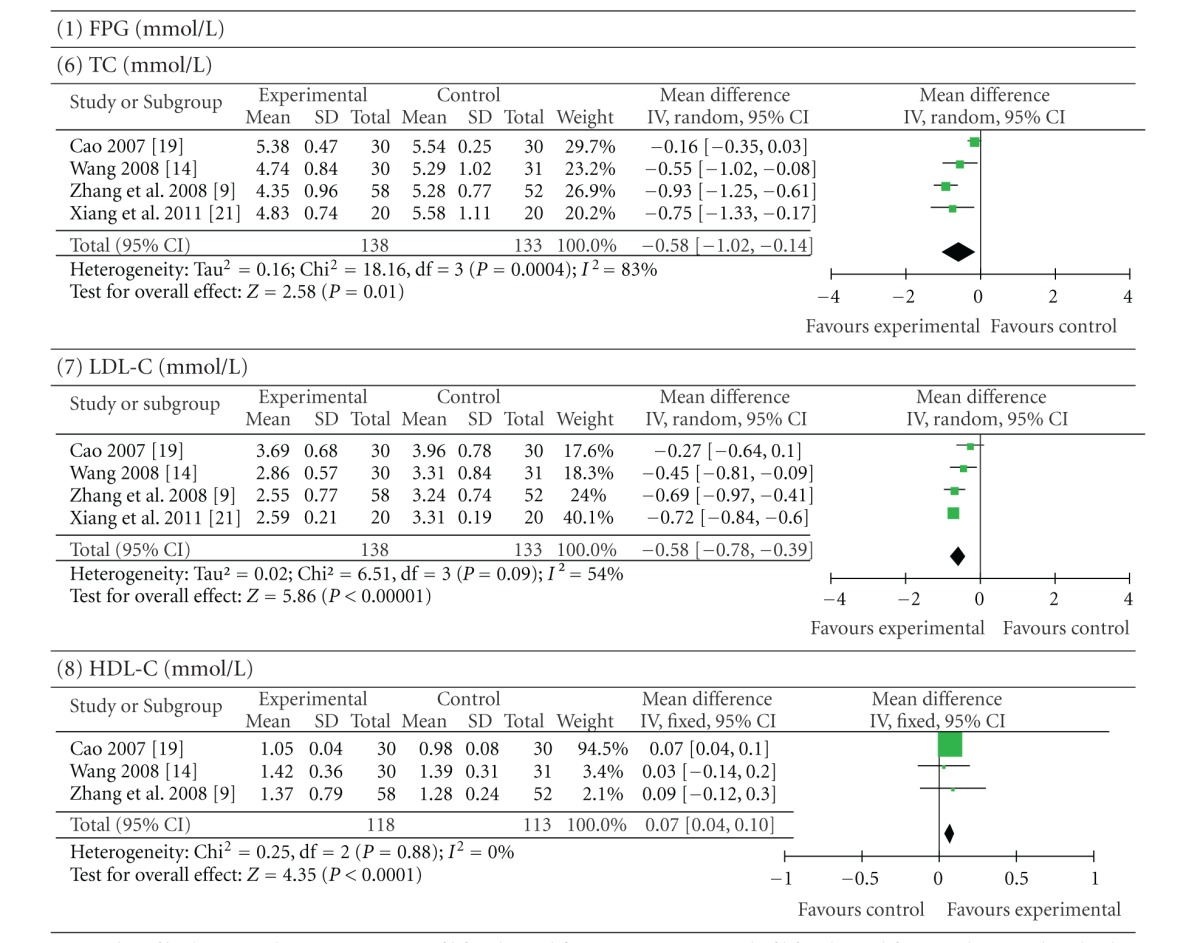

Forest plot of berberine with a cointervention of lifestyle modification versus a control of lifestyle modification alone or plus placebo. FPG: fasting plasma glucose; PPG: postprandial plasma glucose levels; HbA1c: glycosylated haemoglobin levels A1c; FINS: fasting insulin levels; TG: triglycerides; TC: total cholesterol; LDL-C: low-density lipoprotein cholesterol; HDL-C: high-density lipoprotein cholesterol.

**Table 4:** Berberine versus oral hypoglycaemics.

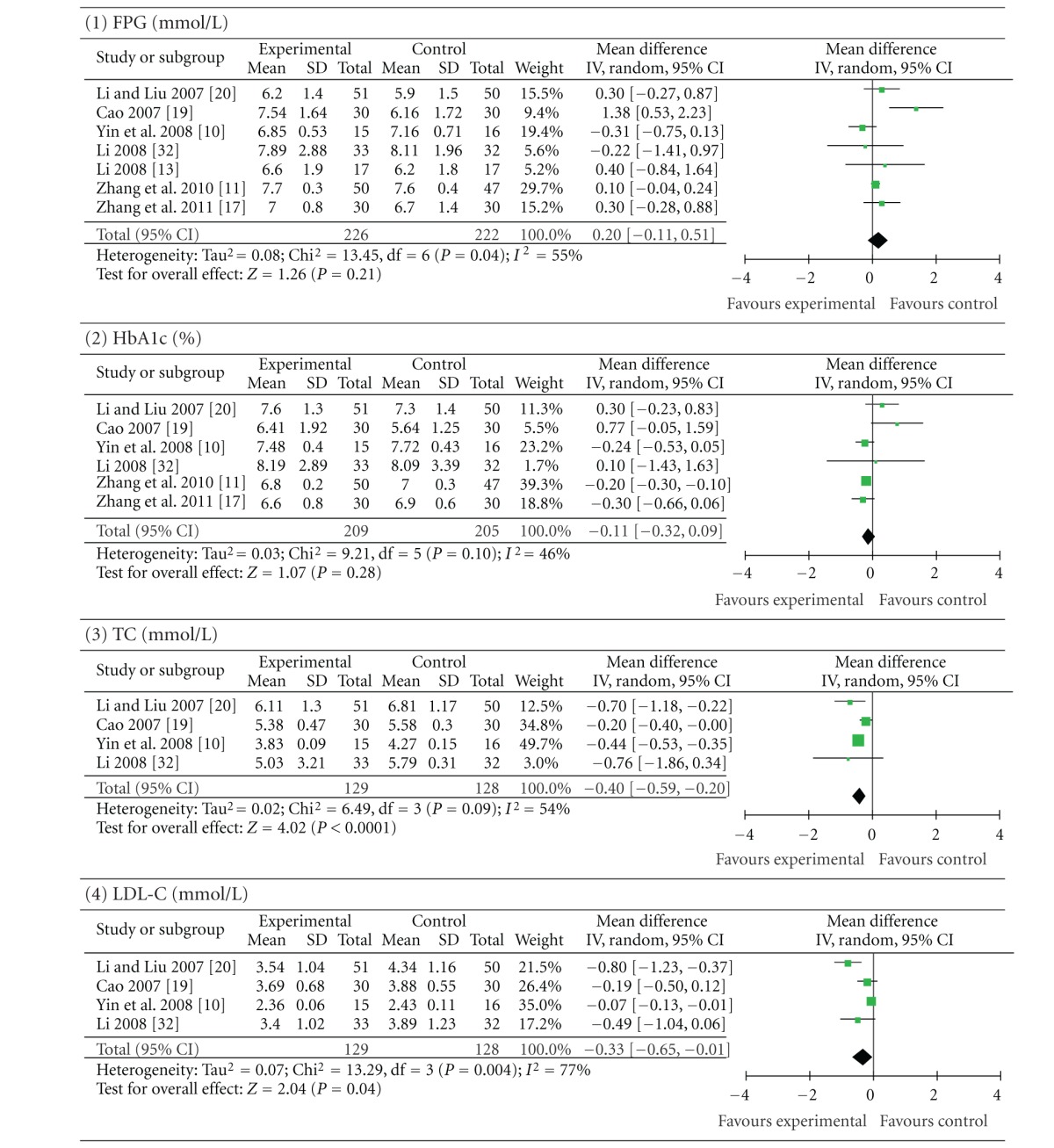

Forest plot of berberine versus oral hypoglycaemics. FPG: fasting plasma glucose; HbA1c: glycosylated haemoglobin levels A1c; TC: total cholesterol; LDL-C: low-density lipoprotein cholesterol.

**Table 5:** Berberine combined with oral hypoglycaemics versus the same hypoglycaemics.

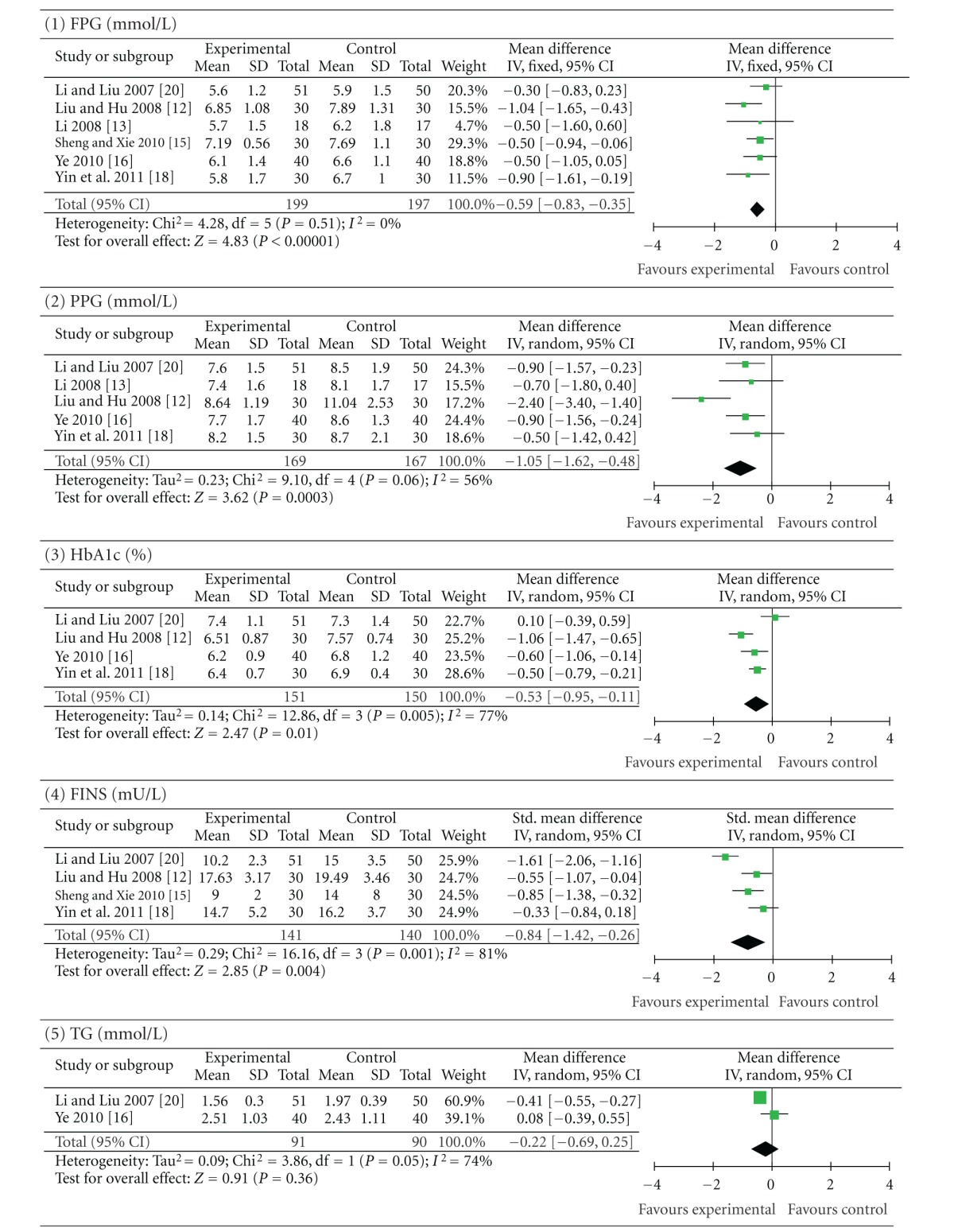 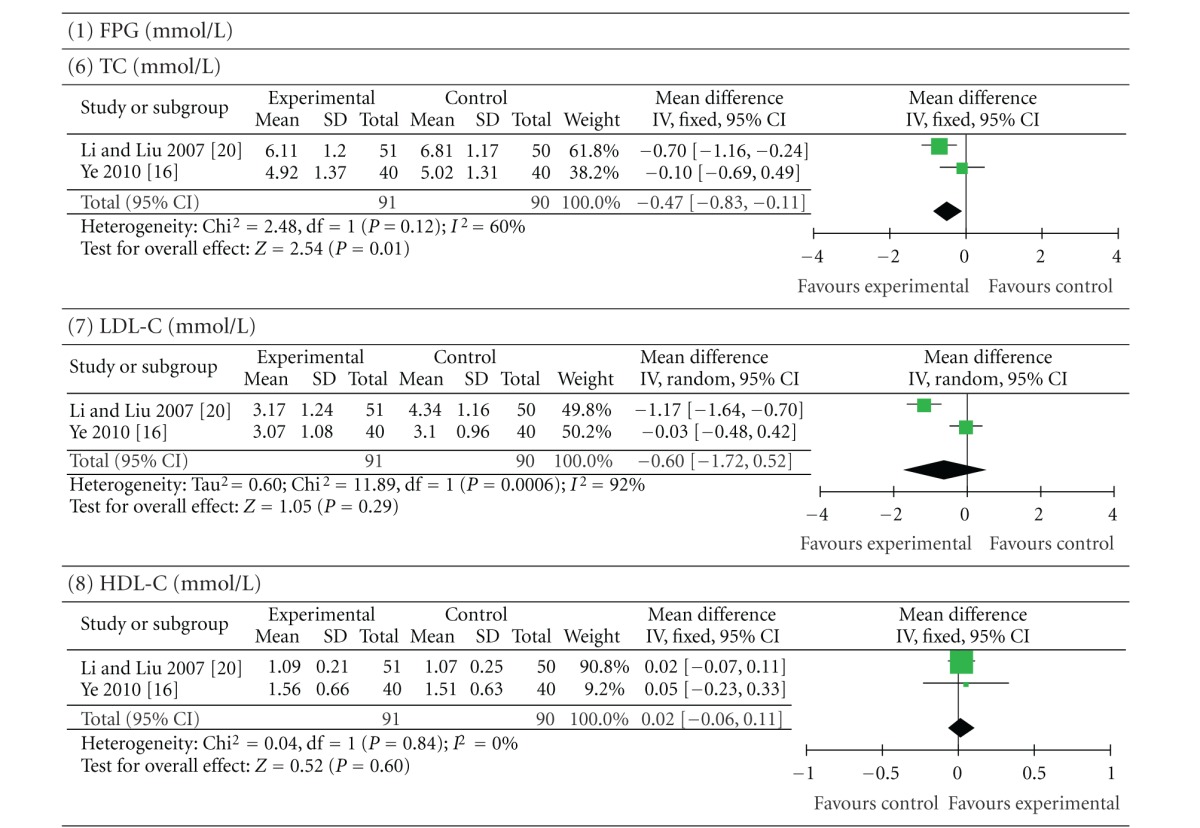

Forest plot of a cointervention of berberine and oral hypoglycaemics versus the same oral hypoglycaemics alone. FPG: fasting plasma glucose; PPG: postprandial plasma glucose levels; HbA1c: glycosylated haemoglobin levels A1c; FINS: fasting insulin levels; TG: triglycerides; TC: total cholesterol; LDL-C: low-density lipoprotein cholesterol; HDL-C: high-density lipoprotein cholesterol.
